# Phloroglucinol Derivatives in Plant-Beneficial *Pseudomonas* spp.: Biosynthesis, Regulation, and Functions

**DOI:** 10.3390/metabo11030182

**Published:** 2021-03-20

**Authors:** Adrien Biessy, Martin Filion

**Affiliations:** Agriculture and Agri-Food Canada, Saint-Jean-sur-Richelieu Research and Development Center, Saint-Jean-sur-Richelieu, QC J3B 7B5, Canada; adrien.biessy@canada.ca

**Keywords:** 2,4-diacetylphloroglucinol, DAPG, *Pseudomonas*, biocontrol, antibiotic

## Abstract

Plant-beneficial *Pseudomonas* spp. aggressively colonize the rhizosphere and produce numerous secondary metabolites, such as 2,4-diacetylphloroglucinol (DAPG). DAPG is a phloroglucinol derivative that contributes to disease suppression, thanks to its broad-spectrum antimicrobial activity. A famous example of this biocontrol activity has been previously described in the context of wheat monoculture where a decline in take-all disease (caused by the ascomycete *Gaeumannomyces tritici*) has been shown to be associated with rhizosphere colonization by DAPG-producing *Pseudomonas* spp. In this review, we discuss the biosynthesis and regulation of phloroglucinol derivatives in the genus *Pseudomonas*, as well as investigate the role played by DAPG-producing *Pseudomonas* spp. in natural soil suppressiveness. We also tackle the mode of action of phloroglucinol derivatives, which can act as antibiotics, signalling molecules and, in some cases, even as pathogenicity factors. Finally, we discuss the genetic and genomic diversity of DAPG-producing *Pseudomonas* spp. as well as its importance for improving the biocontrol of plant pathogens.

## 1. Introduction

Phloroglucinol derivatives are a large class of secondary metabolites widely distributed in plants and brown algae. Over a thousand phloroglucinol derivatives have been characterized to date. As an example, 429 phloroglucinol derivatives have been isolated from the genus *Hypericum* alone [1]. Phloroglucinol derivatives found in plants and brown algae have extremely diverse structures, ranging from the simple grandinol, an acylphloroglucinol produced by several *Eucalyptus* species, to the more complex phlorotannins found in several families of brown algae [2,3]. These compounds often exhibit antiviral, antibacterial and antifungal activity [2]. Phloroglucinol derivatives are also produced by some microorganisms [2,4]. By contrast with the phloroglucinol derivatives found in plants and brown algae, phloroglucinol derivatives of microbial origin are rather simple. Some *Pseudomonas* strains produce 2,4-diacetylphlorglucinol (DAPG) alongside its biosynthetic intermediates monoacetylphloroglucinol (MAPG) and phloroglucinol.

DAPG-producing *Pseudomonas* spp. have received particular attention due to their ability to control numerous soil-borne plant diseases, including take-all of wheat, tobacco black root rot and sugar beet damping-off [4,5]. These bacteria also play an important role in natural disease suppressiveness found in several soils across the world. Besides their presence in the rhizosphere, DAPG-producing *Pseudomonas* spp. are also known to colonize various environment, including the phyllosphere [6], the skin surface of certain amphibians [7] and the surface of marine algae [8]. This review specifically covers rhizosphere-inhabiting DAPG-producing *Pseudomonas* spp.

Several reviews have been previously published on rhizosphere-inhabiting DAPG-producing *Pseudomonas* spp. and their role in take-all decline [4,9]. In this review, we discuss the biosynthesis and regulation of phloroglucinol derivatives in the genus *Pseudomonas*. Then, we tackle the role that DAPG-producing *Pseudomonas* spp. play in several soils naturally suppressive to soil-borne plant diseases. We also discuss the mode of action of phloroglucinol derivatives, which can act as antibiotics, signalling molecules and, in some cases, even as pathogenicity factors. Finally, we discuss the genetic and genomic diversity of DAPG-producing *Pseudomonas* spp. as well as its importance for improving the biocontrol of plant pathogens.

## 2. Genetics, Biochemistry, and Evolution of DAPG Biosynthesis

### 2.1. The Phl Biosynthetic Gene Cluster

**Figure 1:**
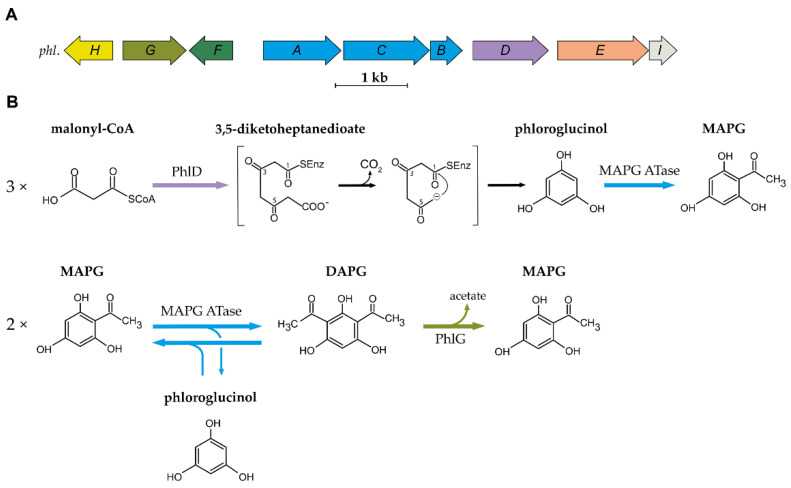
Organization of the 2,4-DAPG biosynthetic cluster and current understanding of the biosynthesis scheme. (**A**) Organization of the *phl* biosynthetic gene cluster found in DAPG-producing *Pseudomonas* spp. (**B**) Current understanding of the biosynthesis and degradation of DAPG in the genus *Pseudomonas*. MAPG ATase is an enzyme multiplex composed of PhlA, PhlB and PhlC units. Abbreviations are as follows: MAPG, monoacetylphloroglucinol; DAPG, 2,4-diacetylphloroglucinol.

Genes involved in DAPG biosynthesis were cloned several times from three different DAPG-producing *Pseudomonas* strains: *Pseudomonas* sp. Q2-87 [10,11], *P*. *kilonensis* F113 [12] and *P*. *protegens* CHA0 [13]. Further characterization of the genomic fragment isolated from *Pseudomonas* sp. Q2-87 led to the description of the so-called *phl* biosynthetic gene cluster (BCG) [14]. Six genes were originally described: four were found to be directly involved in DAPG biosynthesis (*phlABCD*) and the two others were shown to encode a putative permease (*phlE*) and a TetR regulatory protein (*phlF*). Three other genes were later discovered and associated with the BCG: *phlG*, which encodes a hydrolase involved in DAPG degradation [15], *phlH*, which encodes another TetR regulatory protein [15] and *phlI*, which encodes an uncharacterized protein [16,17]. The organization of the *phl* BCG is conserved in all DAPG-producing *Pseudomonas* spp. sequenced to date [18]. The biosynthetic cluster and the current understanding of the DAPG biosynthesis pathway are presented in Figure 1.

### 2.2. Biosynthesis and Degradation of DAPG

The first step in the biosynthesis of DAPG is catalysed by the type III polyketide synthase (PKS) PhlD [14,19]. Type III PKSs are homodimeric enzymes that catalyse the iterative condensation of a starter substrate (usually an acyl-CoA) with several extender substrate units (usually malonyl-CoA) to generate a linear polyketide, which is subsequently cyclized [20]. Bangera and Thomashow [14] proposed that PhlD uses acetoacetyl-CoA as the starter substrate to produce monoacetylphloroglucinol (MAPG), but it was later showed that PhlD produces phloroglucinol from malonyl-CoA instead [19]. While PhlD uses malonyl-CoA as a preferred substrate, it also accepts other starter substrates with an aliphatic chain of C_4_-C_12_ in vitro [21]. The proposed mechanism leading to the formation of phloroglucinol is that PhlD catalyses the iterative condensation of three molecules of malonyl-CoA into 3,5-diketoheptanedioate [19]. This polyketide intermediate undergoes decarboxylation and is subsequently cyclized via a Claisen condensation, leading to the formation of phloroglucinol [19,21].

In the following steps, acetylation of phloroglucinol leads to the formation of MAPG, which is subsequently acetylated into DAPG. MAPG was identified as a putative intermediate in DAPG biosynthesis by Shanahan and colleagues [22], who also found experimental evidence for an enzymatic acetyltransferase activity in cell-free extracts of *P*. *kilonensis* F113. Bangera and Thomashow [14] found that *phlACB* was required for the biosynthesis of MAPG and DAPG, suggesting a role for PhlACB in the production and the acetylation of MAPG. Achkar and colleagues [19] confirmed that the product of *phlACB* catalyses the acetylation of phloroglucinol and MAPG: The addition of phloroglucinol to the culture medium of an *E*. *coli* strain carrying plasmid-localized *phlACB* led to the formation of MAPG and DAPG. Later, Hayashi and colleagues [17] characterized the multimeric enzyme composed of PhlA, PhlC and PhlB units, which was named MAPG acetyltransferase (MAPG ATase). This enzyme, unlike most acetyltransferases described so far, was shown to catalyse C-C bond formation without the use of CoA-activated substrates [17]. The MAPG ATase catalyses the disproportionation of two molecules of MAPG, resulting in the formation of DAPG and the production of phloroglucinol.

The MAPG ATase also catalyses the reverse reaction, yielding two molecules of MAPG from a molecule of DAPG and a molecule of phloroglucinol [17]. This results in an equilibrium where DAPG, MAPG and phloroglucinol are present at quasi-equimolar concentration. Recent studies have provided new insight into the catalytic properties, the mechanism, and the structure of the MAPG ATase [23,24,25,26,27]. This enzyme was shown to use various non-natural substrates as acyl-donor in in vitro experiments [26,27,28]. This is particularly interesting given the fact that the acyl donor involved in MAPG biosynthesis from phloroglucinol remains to be characterized. The crystal structure revealed that PhlACB subunits are arranged in a Phl(A_2_C_2_)_2_B_4_ composition where four PhlB units mediate the binding of two PhlA and two PhlC dimers [23]. Crystal soaking and site-directed mutagenesis experiments suggest that only PhlC units are involved in the acyl transfer reaction [23].

DAPG is degraded by the zinc-dependent hydrolase PhlG [29,30]. PhlG degrades DAPG into MAPG and acetate by cleaving one of the C-C bonds linking the acetyl groups to the phenolic ring [29,30]. This enzyme is highly specific for its substrate DAPG, as it is unable to degrade structurally similar compounds, such as MAPG or triacetylphloroglucinol [29]. The crystal structure of PhlG revealed that it cleaves C-C bonds using a Bet v1-like fold domain, contrary to the alpha/beta fold classically used by hydrolases [30].

### 2.3. Distribution and Evolution of the Phl Biosynthetic Gene Cluster

**Figure 2:**
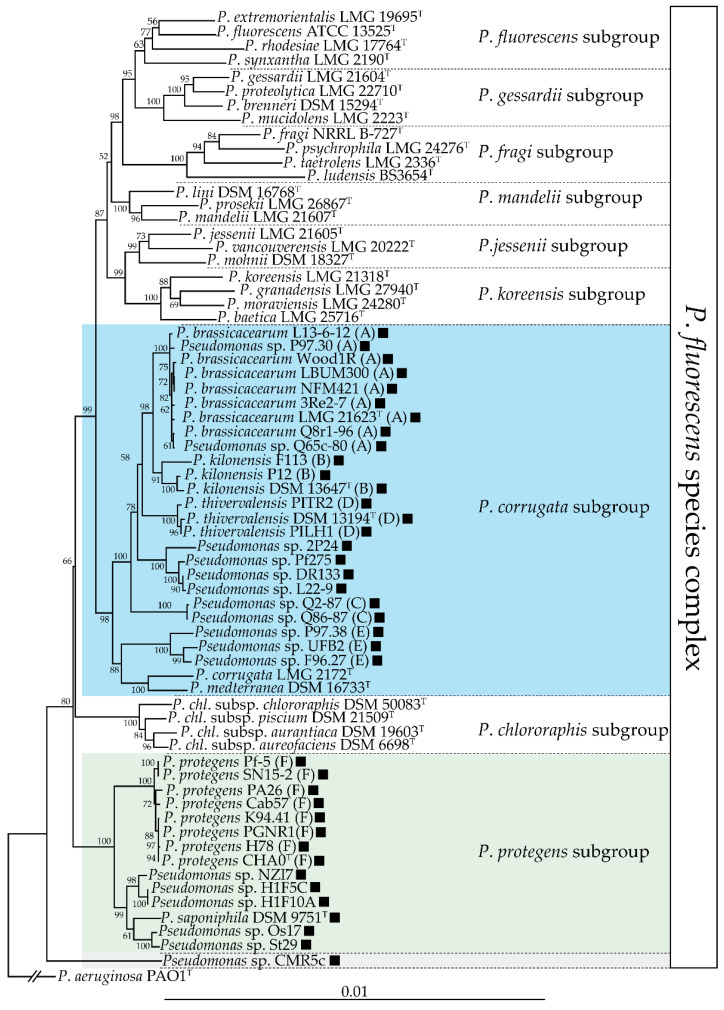
Distribution of the *phl* biosynthetic gene cluster in the *P*. *fluorescens* species complex. This neighbour-joining phylogeny is based on an alignment of the concatenated partial sequences of four housekeeping genes (16s rDNA, *gyrB*, *rpoB*, *rpoD*; 2945 nucleotides total) generated using MUSCLE [33]. The phylogenetic tree was generated using PhyML [34] and the distance matrices were calculated by the Jukes-Cantor method. Bootstrap values over 50% (out of 1000 replicates) are indicated at the nodes. The presence of a black square indicates that that strain harbours the *phl* biosynthetic gene cluster. The three subgroups encompassing DAPG-producing strains are highlighted in color. Letters following the strain names correspond to multilocus phylogenetic groups described by Frapolli and colleagues [35].

The *phl* BCG is mainly found in the *P*. *corrugata* and *P*. *protegens* subgroups of the *P*. *fluorescens* species complex [18], as shown in Figure 2. The *phl* BCG is not present in all the strains belonging to these two subgroups, and its distribution in these two subgroups is patchy [18,31,32].

Almario and colleagues [18] proposed that the *phl* cluster was acquired independently in these two groups and that this cluster was subsequently lost in some lineages of the *P*. *corrugata* subgroup. A recent study reported that the *phl* BCG is present in about half of the genomes sequenced from the *P*. *corrugata* subgroup [32]. Interestingly, this subgroup includes numerous phytopathogenic strains, prominently strains belonging to the species *P*. *corrugata* and *P*. *mediterranea* [36,37]. These phytopathogenic strains do not harbour the *phl* BCG, suggesting that this cluster could have been lost during the transition between commensal and pathogenic lifestyles. The fact that DAPG can trigger induced systemic resistance in some plant species [38,39,40] is an undesirable trait for a plant pathogen, which means that it could have been counter-selected in these lineages. Outside of these two subgroups, the *phl* BCG is also present in several other strains, both inside and outside of the *P*. *fluorescens* species complex [18,41]. The *phl* BCG has also been found outside of the *Pseudomonas* genus: the presence of the *phl* BCG has been reported in three non-pathogenic *Betaproteobacteria*, namely *Pseudogulbenkiania ferrooxidans* EGD-HP2, *Chromobacterium vaccinii* MWU328 and *Chromobacterium* piscinae ND17 [18].

The origin of this cluster remains unclear. Most authors agreed upon the fact that the acquisition of the *phl* BCG in the *P*. *fluorescens* species complex is an ancestral event [16,18,42]. The *phl* BCG might have been acquired separately by the different groups of DAPG-producing *Pseudomonas* spp. [18]. In *Pseudomonas* sp. OT69, the *phl* BCG is embedded in a putative genomic island, suggesting a more recent acquisition by this strain [18]. Kidarsa and colleagues [43] proposed that the *phlACB* genes might have been acquired from an Archaea. Homologs of these three genes are present in a contiguous gene cluster and in the same order in multiple groups of Archaea, where they may play a role in fatty acid metabolism [43].

## 3. Regulation of DAPG Biosynthesis

**Figure 3:**
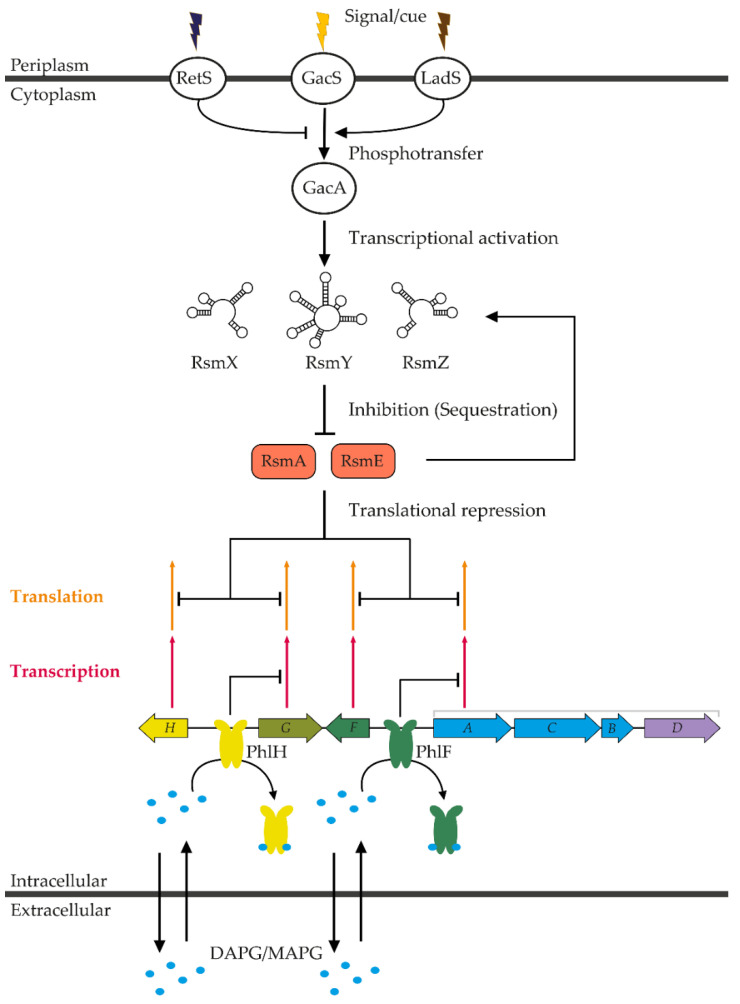
Regulation of DAPG biosynthesis in *Pseudomonas* spp. DAPG production is regulated at the transcriptional level by the TetR repressors PhlH and PhlF, and at the posttranscriptional level via the Gac/Rsm pathway. PhlF binds as a dimer to the operator *phO*, located between *phlF* and *phlA* and represses the expression of *phlABCD*. When DAPG and MAPG concentrations increase, MAPG and DAPG interact with PhlF, dissociating the *phO*-PhlF complex and relieving *phlACBD* transcription. Likewise, PhlH binds to an operator, located between *phlH* and *phlG* and represses *phlG* expression. DAPG and MAPG can dissociate PhlF-DNA binding and relieve *phlG* expression. Upon reception of an environmental cue, GacS activate GacA by phosphotransfer. GacA activates the transcription of several small non-coding RNAs (RsmX, RsmY and RsmZ), which in turn sequester the RNA-binding proteins RsmA/RsmE, relieving *phlABCD* expression.

DAPG production in the genus *Pseudomonas* is regulated by two translational repressors of the TetR family, PhlF and PhlH, and, at the posttranscriptional level, by the Gac/Rsm regulatory network. Figure 3 provides an overview of DAPG regulation in the genus *Pseudomonas*.

### 3.1. Regulation by Translational Repressors of the TetR Family

The *phlF* and *phlH* genes encode two pathway-specific regulators from the TetR family. The TetR family regulators consist of an N-terminal DNA-binding domain and a C-terminal domain [44]. These regulators bind to palindromic repeated sequences localized upstream of the target gene, repressing its expression. In most cases, the C-terminal domain interacts with one or several ligands, which subsequently reduce the ability of the regulator to bind DNA [44]. PhlF has been shown to repress *phlACBD* expression by binding, as a dimer, to an inverted repeated sequence (*phO*) localized downstream of the *phlA* transcriptional start site [45,46]. DAPG and MAPG positively regulate *phlACBD* expression by modulating PhlF activity [15,45]. These two molecules can dissociate the PhlF-*phO* complex in a concentration-dependent manner and prevent further binding of PhlF to *phO* [45]. This suggests that DAPG and MAPG could act as ligands for PhlF. As for PhlH, it regulates the intracellular concentration levels of DAPG by modulating the expression of *phlG* [47]. PhlH binds (likely as a dimer) to the upstream sequence of *phlG* and strongly represses its expression, preventing PhlG-mediated degradation of DAPG [47]. DAPG (and to a lesser extent MAPG) interacts physically with PhlH and can dissociate the PhlH-DNA complex, releasing the PhlH-mediated repression of *phlG*.

PhlF and PhlH regulate DAPG biosynthesis and degradation at different stages of growth. PhlF acts as a repressor of DAPG biosynthesis during the early growth stages. This is evidenced by the fact that DAPG production is observed earlier in a *phlF* mutant [15,45,46]. The growth of the *phlF* mutant is, however, reduced in the early growth stages compared to the wild type [46]. This suggests that, by repressing DAPG production in the early stages, PhlF enables DAPG-producing *Pseudomonas* spp. to outcompete and outgrow other microorganisms. On the other hand, PhlH-mediated repression of *phlG* in the early stages of growth is essential for DAPG production, as a *phlH* mutant produces very low levels of DAPG [15,47]. This indicates that PhlH-mediated repression of *phlG* would normally be abolished later in the growth stages. Yann and colleagues [47] proposed that PhlG promotes bacterial growth in a nutrient-limited environment by reducing the resources allocated to DAPG production in the late growth stages. Indeed, a Δ*phlG* mutant exhibited a lower growth rate and cell density in a nutrient-limited medium compared to the wild type, but this difference was not observed in the richer KB medium. Thus, DAPG sequentially promotes its own biosynthesis and degradation by modulating PhlF and PhlH activity. Furthermore, PhlF-mediated repression of *phlACBD* is abolished sooner during growth when compared to the PhlH-mediated repression of *phlG*. In this regard, we believe that PhlF and PhlH might have differential binding affinities, either for DNA or for MAPG/DAPG to explain this discrepancy. Other factors could also modulate PhlF and PhlH activity.

### 3.2. Regulation by the Gac/Rsm Regulatory Network

The Gac/Rsm signal transduction pathway is well conserved in *Gammaproteobacteria* and regulates, at the posttranscriptional level, the production of several antibiotics, such as hydrogen cyanide, pyoluteorin, phenazine and DAPG [48,49]. The GacA/GacS two-component system governs a complex signal transduction pathway, which involves small non-coding regulatory RNAs (RsmX/Y/Z) and translational repressors (RsmA and RsmE). A *gacA* or *gacS* mutant is unable to produce DAPG [49,50], indicating that GacA/GacS positively regulates DAPG production. The GacA/GacS two-component system is composed of a membrane-bound sensor kinase (GacS) and a cytosolic cognate response regulator (GacA). The Gac/Rsm transduction cascade is initiated by the reception of a signal, which remains to be characterized [51]. GacS undergoes autophosphorylation and subsequently activates GacA by phosphotransfer. Two membrane-bound sensor kinases, LadS and RetS, modulate GacS activity by influencing its phosphorylation state. LadS stimulates GacS activity whereas RetS negatively regulates GacS activity [51,52,53,54]. Notably, RetS was shown to directly interact with GacS [55] to negatively regulate its activity at 35 °C, preventing the production of antibiotics [53]. Upon activation by GacS, GacA upregulates the expression of three small non-coding RNAs, RsmX, RsmY and RsmZ [56]. GacA activates *rsmXYZ* expression by interacting with a conserved palindromic upstream activating sequence (UAS) present in the promoter of *rsmXYZ* [57]. Activation of *rsmXYZ* expression also requires other transcriptional activators that may interact with phosphorylated GacA [57]. These three small non-coding RNAs have a high affinity for the translational repressors RsmE and RsmA [56,58]. RsmA and RsmE are RNA-binding proteins from the RsmA/CsrA family that bind to specific structures located near the ribosome binding site in the leader sequence of target mRNAs, preventing ribosome binding and promoting mRNA decay [50,59,60,61]. A typical hexaloop structure is present in the leader sequence of the *phlA* mRNA and this sequence was shown to be recognizable by RsmA/RsmE [62]. Upon activation of GacA, RsmA and RsmE are sequestered by RsmXYZ, relieving translational repression of target mRNAs [63,64].

The GacS/GacA signal transduction pathway is influenced by the population density and/or the nutritional state of the cells [54,56,57,65]. The expression of *rsmXYZ* increases at the end of the exponential growth phase [56,57,66]. This could originate from the accumulation of a signal in the medium, that activates the Gac/Rsm regulatory network [56,57,66]. It could also be the result of the depletion of the nutrients present in the growth medium, which activates secondary metabolism. The alarmone guanosine tetraphosphate (ppGpp), a signal molecule produced under nutrient limitation, was precisely shown to stimulate the Gac/Rsm signal transduction pathway [54].

### 3.3. Co-regulation of DAPG and Pyoluteorin Production

Several strains belonging to the *P*. *protegens* subgroup have been reported to produce the phenolic antibiotic pyoluteorin [67,68] in addition to DAPG and MAPG. The amounts of DAPG and pyoluteorin being produced have been shown to be inversely correlated [15,67]. This has been especially demonstrated in a CHA0 mutant impaired in pyoluteorin production, which overproduced DAPG and MAPG [69], suggesting a co-regulation between both biosynthetic pathway. In addition, some medium were shown to favour either DAPG or pyoluteorin production [67]. DAPG and pyoluteorin both act as autoinducers of their own biosynthesis [15,70] and the addition of pyoluteorin has been shown to repress *phlA* expression and DAPG production [15,70]. The effect of phloroglucinol derivatives on pyoluteorin production is, however, more complex. The addition of high concentrations of phloroglucinol derivatives (DAPG or phloroglucinol) was shown to repress the expression of genes involved in pyoluteorin production [43,70]. Phloroglucinol production by PhlD was shown, however, to be essential for pyoluteorin production, as a Δ*phlD* mutant was unable to produce pyoluteorin [43]. The addition of low concentrations of exogenous phloroglucinol restored pyoluteorin production in the Δ*phlD* mutant [43], suggesting that small quantities of phloroglucinol are required for pyoluteorin biosynthesis. Interestingly, pyoluteorin-producing *P*. *aeruginosa* strains do not produce DAPG, but they harbour a *phlD* gene adjacent to the pyoluteorin gene biosynthetic cluster, which allows them to produce phloroglucinol [43]. More recently, Yan and colleagues [71] found that phloroglucinol is the substrate of the FADH_2_-dependent halogenase PltM, which converts phloroglucinol into chlorinated phloroglucinol. Chlorinated phloroglucinol activates, probably via the pathway-specific regulator PltR, the expression of pyoluteorin biosynthetic genes [71]. This co-regulation mechanism likely attenuates the metabolic cost of producing several antibiotics, while still providing the option for the bacteria to produce one or the other depending on the situation.

### 3.4. Environmental Factors Influencing DAPG Production

Several environmental factors can influence DAPG production by plant-beneficial *Pseudomonas* spp. First, the type of carbon source available can greatly affect DAPG production. For example, the presence of sucrose, fructose, galactose, or mannitol promotes DAPG production by *P*. *kilonensis* F113, while the presence of glucose and sorbose negatively impacts its production [22,72]. On the other hand, glucose promotes DAPG production in *P*. *protegens* CHA0 and in several other DAPG-producing strains [73], indicating that carbon sources have differential effects on DAPG production depending on the strain genotype. Furthermore, the presence of specific metabolites, such as fusaric acid, can also reduce DAPG production. Fusaric acid is a mycotoxin produced by several *Fusarium* species, including *Fusarium oxysporum* [74]. Fusaric acid was shown to strongly repress DAPG production by plant-beneficial *Pseudomonas* spp. both in vitro and in the rhizosphere of tomato and wheat [15,75,76]. Since an isogenic mutant lacking the pathway-specific repressor PhlF was found insensitive to fusaric acid, it was determined that this mycotoxin likely acts via the modulation of PhlF activity [15,75]. Finally, plants can also influence DAPG production by plant-beneficial *Pseudomonas* spp. colonizing the rhizosphere in several ways. For example, Jousset and colleagues used a split-root system to demonstrate that infection by *Pythium ultimum* resulted in a change in root exudate composition, which leads to an increase in DAPG production by *P*. *protegens* CHA0 colonizing the rhizosphere of the infected plant [77]. Recently, two plant flavonoids, apigenin and phloretin, were shown to repress DAPG production by rhizosphere-inhabiting *Pseudomonas* sp. 2P24 [78]. These two metabolites promote *phlG* expression by modulating PhlH-mediated repression, which ultimately results in the degradation of DAPG.

## 4. Role of DAPG-Producing *Pseudomonas* spp. in Natural Soil Suppressiveness

### 4.1. Take-All Decline

Take-all is an important root disease of wheat caused by the ascomycete *Gaeumannomyces tritici* (formerly *Gaeumannomyces graminis* var *tritici*). This soil-borne pathogen primarily infects wheat, but it can also cause root rot in other *Poaceae*, such as triticale, barley and rye [79,80]. Because of its relative inability to survive for a long period of time in the soil without a host, growing a non-host crop, such as oats, for one or two years can effectively control this disease [81]. An alternative to crop rotation is to grow wheat and barley continuously, which leads to a spontaneous decline in take-all occurrence and severity over time, a phenomenon known as take-all decline (TAD). TAD represents one of the best examples of induced specific suppression, and this field phenomenon occurs across the world [4,82]. It is defined as "the spontaneous decrease in the incidence and severity of take-all that occurs with monoculture of wheat or other susceptible host crops after one or more severe outbreaks of the disease" [82]. Take-all decline is usually achieved after 4–6 years of monoculture of wheat and barley in the same fields [82]. The suppressiveness associated with TAD soils can be transferred to conducive soils by mixing a small amount of TAD soil with conducive soil. TAD suppressiveness can be reduced by growing a non-host plant (oats) and it can be eliminated by soil pasteurization/fumigation [82].

The role that DAPG-producing *Pseudomonas* spp. play in the natural suppressiveness associated with TAD soils was demonstrated in a series of experiments conducted with several TAD soils from the Washington State (USA) and the Netherlands. These soils were compared with local conducive soils [83,84]. Several lines of evidence indicate that DAPG-producing *Pseudomonas* spp. play a preponderant role in take-all decline. First, DAPG-producing *Pseudomonas* spp. were detected in TAD soils at population density greater than 10^5^ CFU per gram of root, which represents the threshold required for take-all control under controlled conditions [83,84,85]. DAPG-producing *Pseudomonas* spp. were not, however, detected in conducive soils or were detected at population densities below the threshold required for the control of take-all [83,84,85]. Secondly, the introduction of a DAPG-producing *Pseudomonas* strain in a conducive soil at sufficient levels resulted in take-all control equivalent to the natural disease suppression found in TAD soils [83,84]. By contrast, the introduction of a mutant impaired in DAPG production did not confer to the conducive soil a level of disease suppression equivalent to TAD soils [84]. Finally, DAPG was isolated from the rhizosphere of wheat colonized by DAPG-producing *Pseudomonas* spp. [86], and the take-all pathogen was shown to be highly sensitive to DAPG [87].

### 4.2. Natural Soil Suppressiveness to Tobacco Black Root Rot

Several morainic soils located in the Swiss region of Morens are naturally suppressive to tobacco black root rot [88,89], a disease caused by the fungus *Thielaviopsis basicola*. By contrast with take-all decline, which is induced by monocultures of wheat and barley, suppressiveness to tobacco black root rot can be found in soils with different cropping history (monoculture or crop rotation) [89]. The natural suppressiveness found in the Morens soils can be eliminated by heat treatment [88], demonstrating its microbial origin. Several DAPG-producing *Pseudomonas* strains were isolated from these suppressive soils, including the model strain *P*. *protegens* CHA0 [88]. When inoculated, strain CHA0 was able to control tobacco black root rot in 36 out of 39 conducive soils [88]. DAPG-producing *Pseudomonas* spp. were found to reach high population densities in the rhizosphere of tobacco plants grown in suppressive soils [90,91]. However, they were detected at similar population levels in conducive soils [90,92]. This suggests that, contrary to take-all decline, disease suppression does not originate from the buildup of DAPG-producing *Pseudomonas* spp. populations in the rhizosphere. Nevertheless, several genotypes of DAPG-producing *Pseudomonas* spp. were exclusively found in the suppressive soils [90,91], suggesting that differences in the population structure of DAPG-producing *Pseudomonas* spp. might explain the differences in suppressiveness between conducive and suppressive soils. However, this conflicts with the finding that DAPG-producing *Pseudomonas* strains isolated from conducive soils protected tobacco roots to a similar extent as isolates from the suppressive soils did [93]. The conducive and suppressive soils of the Morens region have different geological origins and compositions. Indeed, vermiculite is the predominant clay mineral in the morainic suppressive soils, while illite is the predominant one in conducive soils. Several studies have demonstrated that DAPG-producing *Pseudomonas* spp. protected tobacco roots to a better extent in vermiculite soils [94,95]. Indeed, the presence of vermiculite instead of illite is associated with a higher iron availability and a higher *phlA* expression [95]. Lastly, other plant-beneficial microbial strains may be involved and act in concert with DAPG-producing *Pseudomonas* spp. to control tobacco root rot [89,96].

### 4.3. Role of DAPG-Producing Pseudomonas in Other Suppressive Soils

In addition to soils suppressive to take-all and tobacco black root rot, DAPG-producing *Pseudomonas* spp. were isolated from other suppressive soils across the world. Numerous genotypes of DAPG-producing *Pseudomonas* spp. were isolated from the rhizosphere of pea plants grown in soils suppressive to Fusarium wilt [97], suggesting that they could play a role in the natural suppressiveness. DAPG-producing *Pseudomonas* spp. were also found in the rhizosphere of flax and tomato grown in the natural suppressive soils of Châteaurenard, in France [98]. However, they were also found in the conducive soils of Carquefou (France) at similar population levels and probably play a minor role in the suppression of Fusarium wilt.

## 5. Mode of Action

### 5.1. Direct Inhibition of Soil-Borne Plant Pathogens

Phloroglucinol derivatives have been shown to inhibit the growth of numerous bacterial, fungal and oomycete soil-borne pathogens, including the fungal ascomycetes *Gaeumannomyces tritici* and *Thielaviopsis basicola*, the oomycete *Pythium ultimum*, the Gram-negative bacterium *Pectobacterium atrosepticum* and the Gram-positive bacterium *Clavibacter michiganensis* subsp. *michiganensis* [10,12,13,99,100]. Plant pathogens and even different isolates of the same pathogen display differential sensitivity to DAPG [13,87]. Phloroglucinol derivatives produced by plant-beneficial *Pseudomonas* spp. accumulate in the rhizosphere [13,86,101,102,103] at concentrations up to several micrograms per gram of root. DAPG is, however, rapidly degraded in the rhizosphere [103], suggesting that root-associated microcolonies of plant-beneficial *Pseudomonas* spp. actively maintaining DAPG production is required. Plant-associated *Pseudomonas* spp. only colonize a small portion of the root surface [104], which means that, locally, DAPG concentration is likely to be sufficient to inhibit soil-borne plant pathogens. Most reports indicate that, among phloroglucinol derivatives, DAPG is more active than MAPG, whereas DAPG and MAPG are more active than phloroglucinol [105,106]. Like other antibiotics, the antimicrobial activity of phloroglucinol derivatives is strongly influenced by the pH and is higher at lower pH [105]. This could originate from the fact that at high pH, DAPG is likely to be deprotonated, which could impair its capacity to cross biological membranes.

Several studies have examined the effect of phloroglucinol derivatives on the physiology of oomycetes and fungi. Exposure of the oomycete *Pythium ultimum* to DAPG led to the inhibition of zoosporogenesis, zoospore motility and zoospore germination [105], several processes crucial for pathogenesis. The various structures produced by *P*. *ultimum* during its life cycle greatly differ in their sensitivity to DAPG, with zoospores being the most sensitive. Indeed, exposure to DAPG at a concentration as low as 3.2 ng mL^−1^ disintegrated most zoospores [105]. By contrast, vegetative mycelium could tolerate DAPG concentration in the range of 10–20 µg mL^−1^, suggesting several tolerance mechanisms. Microscopy studies of the hyphal tips of *P*. *ultimum* revealed that exposure to DAPG provoked several structural changes, including alteration of the plasma membrane, vacuolization and cell content disintegration [105]. DAPG also inhibits zoosporogenesis and zoospores motility in *Plasmopara viticola* and *Aphanomyces cochlioides* in a dose-dependent manner [106]. The main mode of action of DAPG likely resides in its capacity to act as a proton ionophore, dissipating the proton gradient across the mitochondrial membrane [107,108,109], which lead to the loss of mitochondrial function and the inhibition of growth. Long exposure to DAPG can generate oxidative stress with the production of superoxide and hydrogen peroxide [110], which is similar to the effect that long exposure to other uncoupling agents produces [109].

DAPG exhibits high antimicrobial activity against several bacteria, including the Gram-positive model *Bacillus subtilis* and the Gram-negative plant pathogen *Pseudomonas syringae* [13]. Conversely, it is quite inefficient against *Pseudomonas aeruginosa* and *Pseudomonas fluorescens* strains [13,111]. This discrepancy could originate from differential membrane permeability and/or the presence of detoxifying efflux pumps. It has been proposed that DAPG targets the bacterial envelop [111], but little is known about the mode of action of DAPG towards bacteria. At subinhibitory concentrations, DAPG has been shown to reduce biofilm and spore formation in *B*. *subtilis* [112].

### 5.2. DAPG in Plant-Bacteria Interaction

DAPG-producing *Pseudomonas* spp. can trigger induced systemic resistance (ISR) in *Arabidopsis thaliana* against *Hyaloperonospora parasitica* [38] and *Pseudomonas syringae* pv. *tomato* [39]. In these studies, the authors demonstrated the role of DAPG in the ISR-eliciting activity by using strains impaired in DAPG production. These strains were unable to elicit ISR and to prime the plants against the incoming infection with a pathogen. By contrast, DAPG alone was sufficient to replicate the ISR-eliciting activity of the wild type strains. In an elegant study, Chae and colleagues [40] used transgenic *A*. *thaliana* plants overexpressing *phlG* and found that these plants were rendered insensitive to DAPG-mediated ISR. On the wild type, DAPG was able to induce ISR against *P*. *syringae* pv. *tomato* and *Botrytis cinerea* [40]. In these three studies, DAPG-mediated ISR relied on the ethylene/jasmonate signalling pathway.

Besides its beneficial role in pathogen suppression, DAPG was also shown to be harmful to plants in several cases. High concentrations of DAPG were shown to inhibit plant growth and seed germination in several plant species, including wheat and tomato [13,103]. DAPG also alters root architecture in tomato seedlings, inhibiting primary root growth and stimulating lateral root production [113]. In addition, several DAPG-producing *Pseudomonas* strains were shown to act as minor plant pathogens when inoculated at high population densities. *P*. *brassicacerarum* Q8r1-96 reduced the germination of wheat seeds and caused lesions on wheat roots [103,114]. Recently, *P*. *brassicacearum* strains Q8r1-96 and L5.1-96 were shown to cause necrosis when injected into immature tomato fruits and tomato stems [115]. A mutant unable to produce DAPG was consistently less virulent to tomato fruits and stems, suggesting that DAPG plays a major role in the ability of these strains to cause necrosis [115].

### 5.3. Phloroglucinol Derivatives as Signalling Molecules in the Rhizosphere

Numerous antibiotics have been shown to induce different responses depending on their concentrations, a concept known as hormesis [116,117,118]. At high concentrations, antibiotics are inhibitory and harmful to numerous microorganisms, but at low concentrations (sub-inhibitory concentrations) the antibiotic influences the expression of numerous target genes, serving as a molecular signal. We previously discussed in this review the role that DAPG and phloroglucinol play in the regulation of DAPG and pyoluteorin biosynthesis. DAPG can induce its own biosynthesis and degradation by modulating PhlF and PhlH activity, in a dose-dependent manner [15,45,47]. Phloroglucinol was shown to be necessary for pyoluteorin biosynthesis at nanomolar concentrations, but at micromolar concentrations, it has a negative impact on pyoluteorin production [43]. Phloroglucinol production also influenced the expression of numerous genes with diverse functions unrelated to pyoluteorin production or regulation [119], reinforcing the role of phloroglucinol as an intracellular signalling molecule.

Phloroglucinol-derivatives can also act as signalling molecules in the rhizosphere. DAPG was shown to act as an interpopulation signal in the rhizosphere, where DAPG produced by a strain was able to enhance the expression of the biosynthetic gene *phlA* in another strain [120]. Similarly, phloroglucinol produced by one strain was shown to influence pyoluteorin production in another strain [119]. Several studies indicate that phloroglucinol derivatives could be involved in interspecies signalling. The ISR-eliciting activity of DAPG is one example. Another example is the fact that DAPG production by *P*. *kilonensis* F113 enhances the phytostimulatory effect of *Azospirillum brasilense* Sp245-Rif on wheat by modulating the expression of numerous genes involved in plant growth promotion [121]. Finally, the treatment of conidial germlines of *Neurospora crassa* with DAPG resulted in a transient increase in intracellular Ca^2+^ concentration [107]. Considering the importance of Ca^2+^ homeostasis in plant-microbe interactions, this suggests that DAPG could potentially serve as a signal.

## 6. Genetic and Genomic Diversity of DAPG-Producing *Pseudomonas* spp.

An important diversity of DAPG-producing *Pseudomonas* spp. has been isolated across the world from the rhizosphere of a myriad of plants [4,122,123]. The genetic diversity of DAPG-producing *Pseudomonas* spp. has been studied by various methods, including genomic fingerprinting using BOX/ERIC-PCR [97,124,125], restriction fragment length polymorphism of *phlD* [97,123,125,126,127,128] and phylogenetic analysis of *phlD* [127,129]. This led to the description of 22 genotypes (genotypes A-Q, R, S, T, PfY, PfZ) of DAPG-producing *Pseudomonas* spp. [4]. It is noteworthy that several genotypes are often present in the same field [93,97,98,124,125]. As an example, 5 distinct genotypes (B, C, D, E and F) were isolated from the rhizosphere of wheat grown in the Quincy soils (Washington State, USA) [124]. The phylogeny of DAPG-producing *Pseudomonas* spp. was also studied using multi-locus sequence analysis [35], leading to their classification into six distinct phylogenetic groups, with groups from A to E corresponding to lineages from the *P*. *corrugata* subgroup and group F corresponding to lineages from the *P*. *protegens* subgroup [18,35,42]. Several studies indicate that DAPG production also occurs outside of these six phylogenetic groups [18,130].

The genomes of numerous DAPG-producing *Pseudomonas* strains have been sequenced and, as of today, 151 genomes harbouring the *phl* BCG can be found in the *Pseudomonas* Genome Database [131]. This includes the genomes of well-known strains Pf-5, F113, Q2-87, Q8r1-96 and CHA0 [132,133,134,135]. The analysis of these genomes has enabled the discovery of several unknown phytobeneficial traits. For example, three orphan gene clusters were found in the genome of *P*. *protegens* Pf-5 [132]. This led to the characterization of two novel antibiotics, namely rhizoxin [136] and orfamide [137].

DAPG-producing *Pseudomonas* strains harbour numerous BCG involved in the production of secondary metabolites. Most DAPG-producing strains harbour the *hcnABC* cluster [42,138], which is responsible for the production of hydrogen cyanide (HCN) [139]. HCN was shown to contribute to the suppression of tobacco black root rot when produced by *P*. *protegens* CHA0, as a mutant impaired in HCN production protected tobacco plants less effectively than the wild type did [140]. Likewise, DAPG and HCN were both shown to contribute to the suppression of bacterial canker of tomato, a disease caused by *Clavibacter michiganensis* subsp. *michiganensis* [100]. Several strains from the *P*. *protegens* subgroup harbour numerous BCG involved in the production of secondary metabolites. For example, *P*. *protegens* Pf-5 can produce six metabolites toxic to various oomycetes, fungi, and bacteria: DAPG, hydrogen cyanide, pyoluteorin, pyrrolnitrin, rhizoxin and orfamide [132,137]. The diversity of phytobeneficial traits present in the different DAPG-producing strains may act in synergy to control various plant pathogens or it could expand the biocontrol range by controlling plant pathogens less susceptible to DAPG-mediated inhibition.

The diversity of DAPG-producing *Pseudomonas* spp. is also important, as different genotypes colonize the rhizosphere of various plants with different abilities. For example, strains from genotype D (which includes strain Q8r1-96) are aggressive colonizers of the wheat root surface and rhizosphere, and represent the most common genotype isolated from the rhizosphere of plants grown in Washington State soils that are suppressive to take-all [141]. Genotype K (which includes *P*. *kilonensis* F113) also colonizes the rhizosphere of wheat to a similar extent, while genotype P was not as efficient [142]. However, genotype P outcompetes genotype D and K in the pea rhizosphere [142]. This diversity could be used to select biocontrol agents that are adapted to colonize the rhizosphere of a specific plant species.

## 7. Concluding Remarks

Since the first description of the *phl* BCG by Bangera and Thomashow in 1999, great progress has been made in our understanding of the DAPG biosynthesis pathway. The two core enzymes of this pathway, PhlD and the MAPG ATase have been extensively characterized and the crystal structure of the later has been recently established [23]. These two enzymes have generated a growing interest in their unusual catalytic properties. PhlD has been used to produce high amounts of phloroglucinol in *E*. *coli* strains carrying plasmid-localized *phlD* [143,144,145]. Regarding MAPG ATase, this enzyme is a new biocatalytic tool, which enables C-C bond formation without the need for CoA-activated substrates [23,28]. Despite these advances, several questions remain unanswered regarding the biosynthesis of phloroglucinol derivatives in *Pseudomonas* spp. For example, the identity of the acyl donor leading to the production of MAPG remains unknown. In addition, several authors reported the isolation of various dimers, such as DAPG-DAPG or MAPG-MAPG dimers [4]. Little is known about how they are produced and the role they play in the rhizosphere. Secondly, we have seen in this review that the biosynthesis of DAPG is regulated by the Gac/Rsm signal transduction pathway and by two TetR pathway-specific regulators, PhlF and PhlH. PhlF and PhlH act, to some extent, as sensors of DAPG and MAPG concentration, thereby allowing DAPG-producing *Pseudomonas* spp. to control the amounts of DAPG being produced. Even more interesting is the fact that these two regulators can be influenced by exogenous metabolites, such as fusaric acid [15,75], or by two plant flavonoids, apigenin and phloretin [78]. This suggests that PhlF and PhlH could act as sensors of the rhizosphere environment. Finally, DAPG-producing *Pseudomonas* spp. are efficient biocontrol agents, capable of protecting the plant root system from numerous soil-borne plant diseases. They are present as long-lasting indigenous communities in several agroecosystems, including in fields under wheat and barley monocultures. The fact that *Gaeumannomyces tritici* did not become less sensitive to DAPG after several decades of wheat monoculture [87] is very promising for the extensive use of DAPG-producing *Pseudomonas* spp. as biocontrol agents. This is probably linked to the mode of action of DAPG, which does not target a specific protein.
